# Sensing the world and its dangers: An evolutionary perspective in neuroimmunology

**DOI:** 10.7554/eLife.66706

**Published:** 2021-04-26

**Authors:** Aurora Kraus, Katherine M Buckley, Irene Salinas

**Affiliations:** 1 Department of Biology, University of New MexicoAlbuquerqueUnited States; 2 Department of Biological Sciences, Auburn UniversityAuburnUnited States; Yale School of MedicineUnited States; Yale School of MedicineUnited States

**Keywords:** immunology, nervous system, neuroimmunology, non-model organisms, evolution, metazoans

## Abstract

Detecting danger is key to the survival and success of all species. Animal nervous and immune systems cooperate to optimize danger detection. Preceding studies have highlighted the benefits of bringing neurons into the defense game, including regulation of immune responses, wound healing, pathogen control, and survival. Here, we summarize the body of knowledge in neuroimmune communication and assert that neuronal participation in the immune response is deeply beneficial in each step of combating infection, from inception to resolution. Despite the documented tight association between the immune and nervous systems in mammals or invertebrate model organisms, interdependence of these two systems is largely unexplored across metazoans. This review brings a phylogenetic perspective of the nervous and immune systems in the context of danger detection and advocates for the use of non-model organisms to diversify the field of neuroimmunology. We identify key taxa that are ripe for investigation due to the emergence of key evolutionary innovations in their immune and nervous systems. This novel perspective will help define the primordial principles that govern neuroimmune communication across taxa.

## 1.Introduction: animal nervous and immune systems communicate to sense danger

As multicellular organisms evolved increasing complexity, molecular mechanisms for sensing the environment arose in both the immune and nervous systems. Although the immune and nervous systems are traditionally studied independently, growing evidence suggests shared evolutionary requirements drove integration of both systems at the genomic, molecular, cellular, and tissue levels. In mammals, the immune and nervous systems deploy coordinated mechanisms for pathogen recognition and control. Notably, reciprocal regulation occurs at several layers: neuronal signals fine-tune the strength of immune responses, whereas mechanisms traditionally associated with immunity mediate neural growth, development, and function (Aurora and Olson, 2014; Cardoso et al., 2017; Chu et al., 2020; Foster et al., 2017; Gonzalez-Figueroa et al., 2021; Pinho-Ribeiro et al., 2017; Ramirez et al., 2020; Zigmond and Echevarria, 2019). These bidirectional, complex interactions are the product of millennia of coevolution and lie at the heart of many physiological processes (Chesné et al., 2019; Huh and Veiga-Fernandes, 2020; Jain et al., 2020). Throughout metazoan evolution, the immune and nervous systems have developed into complementary processes to achieve an imperative function: sense the environment and detect danger. As a result, neuroscientists and immunologists continue to uncover molecules with dual functions in both physiological systems (Table 1 and reviewed by Kolosowska et al., 2019; Minnone et al., 2017; Ni and Gilbert, 2017; Stevens et al., 2007; Yang et al., 2016).

**Table 1.** Molecules with dual roles in the immune and nervous systems.

Factors classically associated with immune functions				
Protein		Immune system properties	Nervous system properties	References
Antimicrobial peptides (AMPs)		Secreted by epithelial and phagocytic cells Disrupt microbial membranes leading to destruction of pathogen	Antimicrobial in nervous system niches Control chemotaxis of immune cells and astroglia Mediate iron homeostasis Modulate nerve impulses Implicated in aging and neurodegeneration	Hanson et al., 2019; Lezi et al., 2018; Su et al., 2010; Zasloff, 2002
Cytokines	TGF-β	Produced by all leukocytes Regulates hemocyte proliferation Generally anti-inflammatory Inhibits B cell proliferation Influences development of T_regs_ and T_H_17 cells	Produced by neurons Controls feeding behavior Angio-suppressive roles in the brain Regulates neuronal development and axon outgrowth	Arnold et al., 2014; Arrieta-Bolaños et al., 2012; Eisenstein and Williams, 2009; Hirota et al., 2015; Makhijani et al., 2017; Morishima et al., 2009; Singh and Aballay, 2019; Yi et al., 2010; You et al., 2008; Zheng et al., 2006
	IL-4, IL-13	Induce T_H_2 antiparasitic immunity, tissue repair, allergic responses	Regulate spatial learning and neurogenesis Bias astrocytes and microglia toward M2/neuroprotective states Mediate oligodendrocyte growth and re-myelination	Fallon et al., 2002; Gadani et al., 2012; Kolosowska et al., 2019; McKenzie et al., 1998; Yang et al., 2016; Zhang et al., 2019a
	TNF-α	Pro-inflammatory functions	Expressed in neurons after damage for acute protection Long-term presence in the CNS is associated with decreased proliferation and neurogenesis Alters permeability of the blood-brain barrier Induces changes in sleep behavior	Borsini et al., 2015; Lambertsen et al., 2009; Liu et al., 1994; Takei and Laskey, 2008; Vanderheyden et al., 2018
Complement System Proteins	Complement factors	Opsonize pathogens for activation of innate and adaptive immune cells	Anti-inflammatory roles during CNS infection Regulate synaptic pruning of microglia expressing C3 receptor Regulate adult neurogenesis by causing increased maturation and migration of progenitors in SVZ and dentate gyrus	Hammad et al., 2018; Nonaka, 2001; Rupprecht et al., 2007; Shinjyo et al., 2009; Stevens et al., 2007
	Perforin-like factors	Pore-forming proteins released by cytotoxic leukocytes Form the membrane attack complex	﻿ASTNs and ﻿BRINPs are expressed in CNS and associated with neurodevelopment	Ni and Gilbert, 2017
Pattern Recognition Receptors	Toll-like receptors (TLRs)	Detect extra- and intracellular pathogen and danger associated molecular patterns.	Regulate neuronal development, dendrite/axon growth and synapse formation Recognize neurotrophins Sensitize nociceptive neurons	Chen et al., 2019c; Donnelly et al., 2020; Foldi et al., 2017; Franzenburg et al., 2012; Lemaitre et al., 1996
	Nod-like receptors (NLRs)	Detect intra-cellular pathogen and danger associated molecular patterns.	Immunomodulate glial cells Prevents necrosis of neurons	Gharagozloo et al., 2017
	﻿Peptidoglycan recognition protein LC (PGRP-LC)	Detects peptidoglycan	Controls presynaptic homeostasis	Harris et al., 2015
	Formyl peptide receptors (FPRs)	Expressed on macrophages Detect pathogens and induce inflammation	Vomeronasal sensory neurons receptors	Dietschi et al., 2017
Histamine		Released by mast cells Mediates vasodilation and itch	Modulates neurogenic inflammation and nociceptive inflammation and has been implicated in migraines Induces NGF expression by peripheral nociceptors	Yuan and Silberstein, 2018
**Factors classically associated with neuronal functions**				
Transient receptor potential (TRPs)		Expressed by lymphocytes, dendritic cells, neutrophils, monocytes, macrophages, and mast cells. Cause changes in intracellular Ca^2+^, which influences cell migration, cytokine production, phagocytosis and proliferation	Expressed by distinct subsets of sensory cells Mediate neuronal depolarization and release of CGRP	Alpizar et al., 2017; López-Requena et al., 2017; Parenti et al., 2016
Nerve growth factor (NGF)		Released by mast cells, B lymphocytes. Increases during inflammation Receptor (TrkA) is expressed throughout immune system Transduced NGF signal is anti-inflammatory	Stimulates growth, survival, and differentiation of neurons	Minnone et al., 2017; Pinho-Ribeiro et al., 2017; Takei and Laskey, 2008
Brain-derived neurotrophic factor (BDNF)		Implicated in lymphocyte development and survival	Regulates neuron growth, survival and synapse modulation	Fauchais et al., 2008; Lee et al., 2012; Linker et al., 2015; Schuhmann et al., 2005
Olfactory receptors		Activate pulmonary macrophage motility and CCL2 expression Highly expressed in secondary lymphoid organs	Detect chemical odorants	Heimroth et al., 2020; Li et al., 2013
Calcitonin gene related peptide (CGRP)		An anti-inflammatory cytokine that promotes type 2 immunity Decreases antigen presentation by MHC II, and inflammatory cytokine expression Increases expression of the anti-inflammatory cytokine IL-10 Potent vasodilator Released by T and B lymphocytes	Mediates pain transduction by nociceptors Regulates regeneration of peripheral neurons	Chung, 2017; Kerage et al., 2019; Pinho-Ribeiro et al., 2017; Xu et al., 2019
Substance P		Secreted by microglia, T cells, macrophages, dendritic cells and eosinophils Affects cytokine expression by binding to neurokinin receptor	Neuropeptide involved in nociception and neuroinflammation as well as hypotension and muscle contraction	Mashaghi et al., 2016
Dopamine		Lymphocytes and myeloid cells express the dopamine receptor Enhances lymphocyte chemotaxis and maturation Produced by dendritic cells	Neurotransmitter	Kerage et al., 2019; Matt and Gaskill, 2020
DSCAMs		Acts as a pattern recognition receptor that mediates phagocytosis in arthropods	Regulates axon/dendrite segregation during neuronal development	Goyal et al., 2019; Hattori et al., 2009; Ng and Kurtz, 2020
NCAM/CD56		Present on NK cells, activated T cells and other cytotoxic cell subsets	Neuronal cell migration and synaptic plasticity	Van Acker et al., 2017; Vukojevic et al., 2020
SNARE		Exocytosis of perforins, granzymes, and cytokines	Exocytosis of neurotransmitters	Ramakrishnan et al., 2012; Tang, 2015

Neuroimmune cell units (NICUs) have been described in mammals as discrete anatomical locations where immune and neuronal cells physically interact and regulate tissue physiology and defense (Godinho-Silva et al., 2019). Mammalian neuroimmune interactions are mediated by soluble factors such as neurotransmitters, neuropeptides, and cytokines (Table 1 and reviewed in Trakhtenberg and Goldberg, 2011). Neurons innervate lymphoid organs, thereby influencing immune cell migration and development in mammals (Huang et al., 2021). In turn, specialized immune cells are present throughout nervous tissues where they influence regenerative capabilities as well as regulate pain in response to noxious stimuli (Bloom, 2014; Jung et al., 2017; Pinho-Ribeiro et al., 2017; Huang et al., 2021). Specifically, in mammals, nociceptive neurons in the lung, gut, and skin detect bacteria and release neural mediators that bias the type and magnitude of the immune response toward neuroprotection from infection (Baral et al., 2018; Chiu et al., 2013; Gabanyi et al., 2016; Matheis et al., 2020). Despite the growing appreciation for NICUs in mammals, NICUs are largely unexplored across metazoans, although the soluble molecules and their homologs that mediate communication within NICUs are conserved across animals (Jékely, 2013; Liongue et al., 2016; Mittal et al., 2017; Roch and Sherwood, 2014).

Preliminary work in invertebrate model organisms suggests that cooperation between the nervous and immune systems is a central aspect of animal life that extends beyond mammals. For example, *C. elegans* employ sensory neurons to mediate avoidance behavior in response to pathogenic bacteria and to suppress deleterious innate immune responses (Cao and Aballay, 2016; Hoffman and Aballay, 2019). Several branches of the innate immune system are missing in *C. elegans* and identification of pattern recognition receptors (PRRs) in nematodes is still elusive. Neurons and neurotypical receptors such as G-protein-coupled receptors (GPCRs) appear to compensate for this absence and govern innate immunity in *C. elegans* (Irazoqui et al., 2010; Pujol et al., 2001; Wani et al., 2020; Venkatesh and Singh, 2021). Another example may be Hydra, another basal metazoan with a simple body plan where the TLR signaling pathway appears to have degenerated and neuronally secreted antimicrobial neuropeptides sense and control microorganisms (Augustin et al., 2017; Table 1). Thus, while morphologically simple animals like *C. elegans* and Hydra may not have discrete anatomical associations of immune effector cells and neurons such as mammalian NICUs, their neurons directly regulate tissue physiology and immunity. Of note, both *C. elegans* and Hydra neurons have been mapped in great anatomical detail, and therefore, neuroimmune interactions at these sites may be straight forward to identify. In *Drosophila*, sensory neurons contact hemocytes (a diverse population of macrophage-like cells) in hematopoietic pockets and regulate proliferation, survival, and localization (Cattenoz et al., 2020; Makhijani et al., 2017) perhaps representing small, simple NICUs. Thus, although this field remains largely unexplored, we propose that NICUs are a fundamental aspect of animal physiology and are present in specialized configurations with several degrees of complexity across all metazoans. Given that adaptive immune responses emerged relatively recently (~500 million years ago [mya]), ancient NICUs must have evolved exclusively from the interactions between innate immune cells and neurons or neuroimmune cells (Cooper and Alder, 2006). Despite these hints from non-mammalian model species, the neuroimmunology field still lacks a broader phylogenetic perspective.

Given the functional similarities, frequent crosstalk, and evolutionary overlap between the nervous and immune systems, this review highlights how the tight evolution of immune cells and neurons in a pathogen-laden environment led to neuroimmune cooperation early in metazoan evolution.

## 2.Sensing danger: what came first, immune cells or neurons?

Immune and neural responses are evident even in single-celled organisms, which must constantly sense and respond to the environment. The single-celled protozoan *Stentor coeruleus* exhibits behaviors to environmental cues that are both predictable and can be learned by habituation (Dexter et al., 2019; Tang and Marshall, 2018; Wood, 1970). Furthermore, *S. coeruleus* demonstrates immune capabilities such as wound healing and curation of a microbiome that is separate from the environment (Blauch et al., 2017; Lanzoni et al., 2019). The importance of bacterial symbionts is highlighted by choanoflagellates, which require bacterially derived lipids to create multicellular colonies (Woznica et al., 2016). The origin of multicellularity allowed for the evolution of specialized cell types. As multicellular animals became more complex, the need arose to transmit information among cell types and tissues, ultimately driving evolution of the complex immune and nervous systems present in modern-day mammals. But which appeared first in eukaryotes: immune cells or neurons? Here we propose that a cell with antimicrobial or phagocytic capabilities, whether it was an immune cell or a sensory neuron, predates the emergence of the first in sensu stricto neuronal cell type.

**Figure 1.**
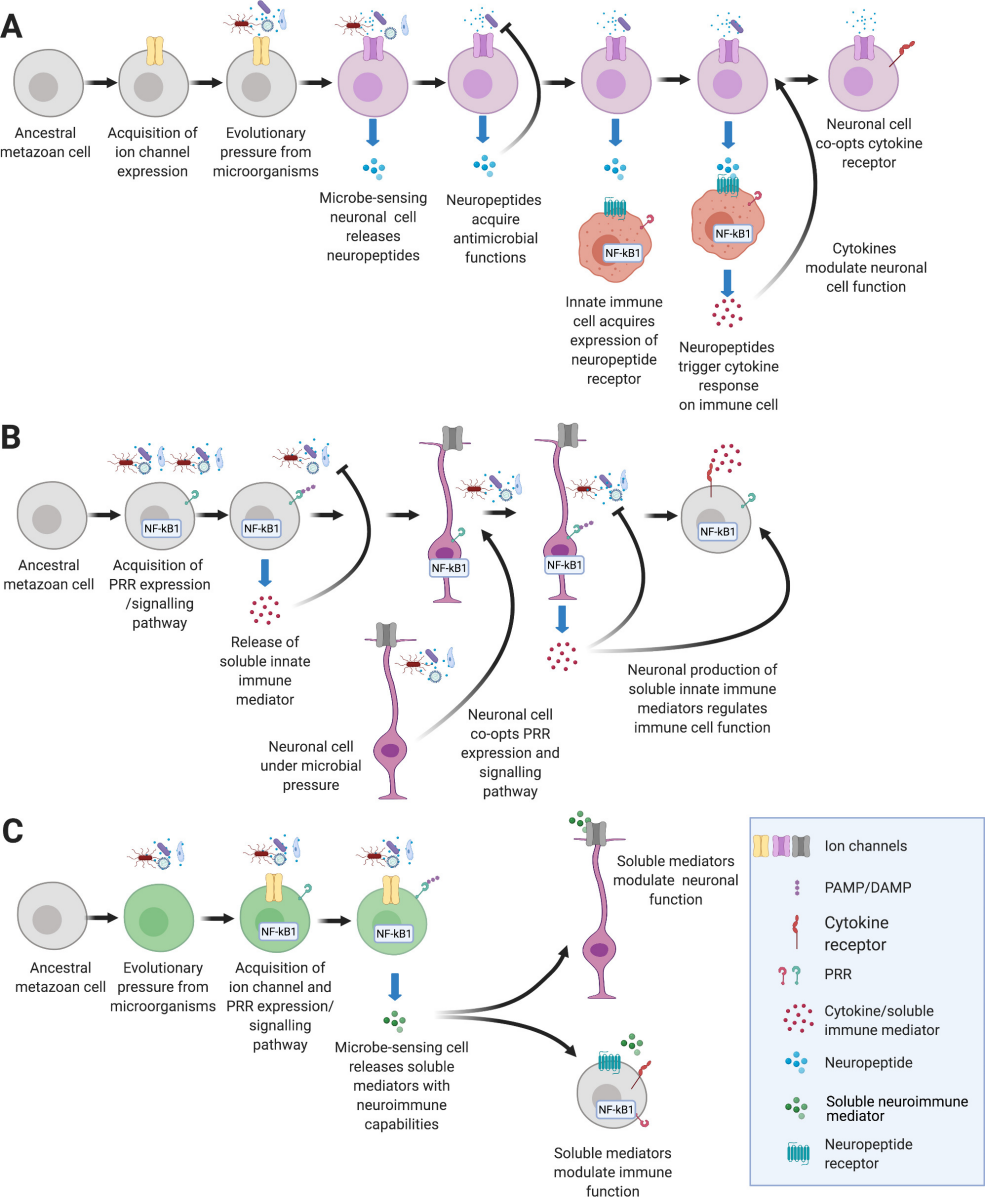
Hypothetical pathways that lead to the emergence of NICUs in early metazoans. (**A**) An ancient metazoan cell under microbial pressure acquires ion channels gaining the ability to detect danger (Elkhatib et al., 2019; Senatore et al., 2016). Eventually neuropeptides that have antimicrobial functions are released in response to microbes (Augustin et al., 2017). Innate immune cells gain neuropeptide receptors that trigger cytokine release (Pinho-Ribeiro et al., 2017). Finally, neuronal cells gain the ability to detect and respond to immune signaling cytokines (Chatterjea and Martinov, 2015; Chu et al., 2020). (**B**) An ancient metazoan cell acquires pattern recognition receptors and gains the ability to detect microbes (Boller and Felix, 2009; Rosenstiel et al., 2009). Eventually soluble immune mediators are released in response that trigger antimicrobial functions (Hanson et al., 2019). Neurons gain immune ligand receptors that trigger neuropeptide release (Lezi et al., 2018). Finally, immune cells gain the ability to detect and respond to neuronally derived neuropeptides (Chu et al., 2020; Foster et al., 2017). (**C**) An ancient metazoan cell under microbial pressure acquires pathogen pattern recognition receptor pathways concurrently with acquisition of ion channels that, under selective pressure gain the ability to detect danger (Tian et al., 2019). Eventually soluble neuroimmune mediators are secreted in response to pathogens modulate functions of neuronal and immune systems.

We propose two hypothetical scenarios in which evolutionary pressure from microbes coerced ancient, conserved molecules like PRRs, soluble factors (i.e., cytokines, chemokines, and neuropeptides), and ion channels, to coordinate communication between neurons and immune cells forming NICUs over 700 mya (Figure 1). This is, in part, based on observations in MyD88-deficient Hydra, demonstraiting that TLR signaling is required for pathogen defense and microbiota colonization and by the discovery of voltage-gated calcium channels in early metazoans known to regulate immune responses to pathogens (Franzenburg et al., 2012; Gupta et al., 2009; Rosenstiel et al., 2009; Senatore et al., 2016). In the first scenario (Figure 1A), an ion channel gains the ability to sense microorganisms, ultimately changing the membrane potential and signaling the cell to release neuropeptides. This phenomenon is highlighted in placozoa where Na^+^ channels sense changes to the microenvironment related to microbes, such as pH (Brunet and Arendt, 2016; Elkhatib et al., 2019; Moran et al., 2015). Over evolutionary time, neuropeptides acquire antimicrobial functions that affect pathogens usually encountered by the cell as revealed in Hydra studies (Augustin et al., 2017). Surrounding cells with immune functions gained expression of neuropeptide receptors, tuning into danger signals from ion channel expressing proto-sensory neuronal cells and releasing cytokines (Satake et al., 2019). In turn, the proto-sensory neuron eventually may gain expression of cytokine receptors to monitor signals from immune cells linking immune ligand receptor binding on neuronal cells’ surface to neuropeptide secretion as reported in studies into pain and itch (Chatterjea and Martinov, 2015; Cunha et al., 2008; Oetjen et al., 2017; Pinho-Ribeiro et al., 2017; Salvador et al., 2021). Together, neuropeptides and cytokines synchronize antimicrobial responses of neuronal and immune cells, forming a simple NICU.

In an alternate, inverse scenario (Figure 1B), an ancestral metazoan cell expresses PRRs and links pathogen associated molecular patterns (PAMPs) to secretion of soluble immune factors such as cytokines and antimicrobial peptides (AMPs). This chain of events is well-studied in PRRs, which are present in plants as well as animals (Boller and Felix, 2009; Rosenstiel et al., 2009). We propose that in a microbe-rich environment, the proto-immune cell releases soluble mediators to defend the host against pathogens and/or sense microbiota. Interestingly, AMPs, which potently destroy bacteria, are lost in the genomes of some fly lineages that live in sterile environments accentuating the importance of microbial pressure in immune repertoire evolution (Choi et al., 2015; Hanson et al., 2019). Notably, AMPs have been shown to regulate sleep and memory (Barajas-Azpeleta et al., 2018; Sinner et al., 2021; Table 1). As explained above, neuronal cells may simultaneously detect danger using prototypical immune receptors to signal the secretion of neurotransmitters which subsequently evolve to reciprocally modulate immune cell function (Cunha et al., 2008; Oetjen et al., 2017; Pinho-Ribeiro et al., 2017).

These two scenarios are likely not mutually exclusive and may have occurred concurrently in early metazoans (Figure 1C). Both PRRs and ion channels are evolutionarily ancient (Rosenstiel et al., 2009; Senatore et al., 2016). Even studies into plants show cooperation of calcium channels and PRRs to regulate innate immunity (Jogawat et al., 2020; Tian et al., 2019). Furthermore, soluble mediators that have been traditionally classified as either neuronal or immune factors are being found to serve both systems (Table 1). We may never glean conclusive evidence as to whether PRRs or ion channels evolved and impelled mutually beneficial communication between early neuronal and immune cells. We postulate on the sequence of events using known interactions from lower metazoans and resulting NICUs from mammals to identify gaps in our current understanding of neuroimmune communication. This novel phylogenetic perspective will help define the elemental principles that govern neuroimmune communication across taxa.

**Figure 2.**
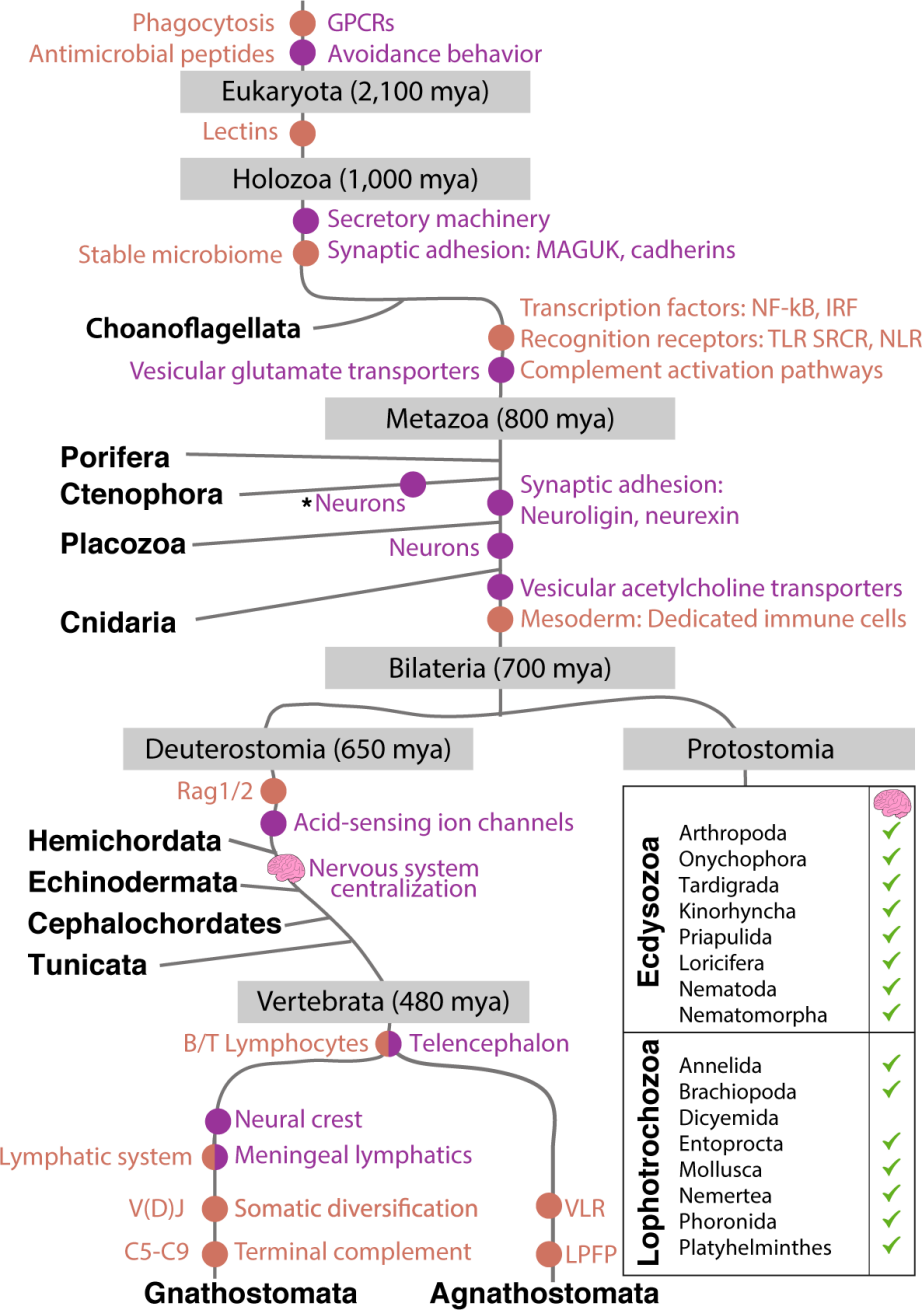
Emergence of the immune and nervous systems predates the emergence of Metazoa. Analysis of genome sequences indicates that much of the cellular machinery involved in extant nervous and immune systems was present in basal metazoan lineages. This includes ion channels, pattern recognition receptors, and antimicrobial peptides (Franzenburg et al., 2012; Rosenstiel et al., 2009; Senatore et al., 2016). The evolutionary origin of significant innovations is indicated by dots (immune system in orange; nervous system in purple). Centralization of nervous systems within bilaterian phyla is indicated by small brain icons. Note CNS structures may be very diverse among invertebrate groups. Asterisk indicates possible independent, convergent evolution of Ctenophore neurons, a current point of debate (Moroz et al., 2014).

The dual roles of ion channels in the nervous and immune systems may also reflect ancient evolutionary pressures (Table 1 and Figure 2). In the calcium-rich ‘primordial soup’, injury to a single-celled eukaryote’s plasma membrane would result in rapid depolarization via environmental calcium influx. Signaling via this swift change in ion concentration links cellular repair as well as endo- and exocytosis to action potentials (Brunet and Arendt, 2016). In multicellular organisms, calcium influxes and depolarization also occur, but result in signals that affect both local and distant cells. Thus, the role of membrane depolarization in cell defense may have predated the need to communicate across an organism.

During infection, not only are microbes present, but the extracellular microenvironment is also chemically altered (e.g., oxygen levels, pH, osmotic pressure) (Alam et al., 2016). Given the concurrence between microbes and chemical factors, it is not surprising that proteins involved in sensing danger evolved to regulate both neural and immune processes (Elkhatib et al., 2019). One example is the transient receptor potential (TRP) channels, which are conserved across Metazoa (Table 1 and Figure 2). TRPs respond to a myriad of environmental cues including chemical, mechanical, and thermal stimuli and are expressed in both neurons and immunocytes (López-Requena et al., 2017; Lynagh et al., 2018; Nilius and Owsianik, 2011; Parenti et al., 2016). TRPs have been linked to avoidance behavior in *C. elegans*, in which TRPV proteins monitor oxygen levels to avoid hyperoxia and specific pathogens (Chang et al., 2006; Singh and Aballay, 2019). Similarly, in planaria, TRPA1 directly mediates extraocular light avoidance behavior (Birkholz and Beane, 2017). Finally, TRPs mediate inflammatory responses by controlling neuronal release of neuropeptides, such as calcitonin gene-related peptide (CGRP), which subsequently regulates immune response and cytokine secretion (Khalil et al., 2018; Marics et al., 2017; Nagashima et al., 2019; Yaraee et al., 2003; Zhang et al., 2018; Table 1). The reciprocal relationship between neuronal and immune proteins will be explored here in greater depth in Section 8. Furthermore, the use of immune and neuronal building blocks by basal metazoans prompted us to question the origins and evolutionary history of NICUs, as discussed in the next section.

## 3.Basal metazoans and the origins of NICUs

The field of neuroimmunology has largely been dominated by a biomedical perspective fueled by the powerful therapeutic interventions that can stem from understanding neuroimmune communication in health and disease. However, the intricacies of neuroimmune communication can be best disentangled when we investigate organisms with reduced body plan complexities and limited numbers of cell types. NICUs have been described in detail in mammals (see review by Godinho-Silva et al., 2019) but remain uncharacterized in most Metazoans.

The ability to respond to bacterial cues and maintain a stable microbiome is ancient within holozoans, including the single-celled choanoflagellate (*Salpingoeca rosetta*) and porifera (*Aplysina aerophoba*) (Pita et al., 2018b; Pita et al., 2018a). Genome surveys have uncovered molecular signatures of both vertebrate immune and nervous systems in the earliest single-celled eukaryotes, including ion channels and post-synaptic scaffolding proteins (Csaba, 2016; Hoffmeyer and Burkhardt, 2016; Vig and Kinet, 2009). The presence and absence of genes encoding these proteins can inform our evolutionary understanding of cells exhibiting immune and neural functions (Fernández and Gabaldón, 2020). Although the relationships between the four major Metazoan lineages that predate Bilateria – Porifera (sponges), Ctenophora (comb jellies), Placozoa and Cnidaria (corals and sea anemones) – remain under debate, identifying cells with immune and nervous functions in these lineages should reveal fundamental aspects of neuroimmune communication (Nielsen, 2019).

Both poriferans and placozoans lack discrete neural structures, whereas cnidarians rely on nerve nets consisting of neurons that resemble those found in bilaterians (Figure 2). In ctenophores, cells with neural morphology form diffuse networks, although the absence of typical metazoan neurotransmitters and synaptic proteins suggests that this nervous system arose via convergent evolution (Moroz, 2015; Moroz et al., 2014). Interestingly, ctenophores possess specialized prey capture cells known as colloblasts, which appear to share an evolutionary progenitor with metazoan neurons (Babonis et al., 2018).

Organisms within Porifera, the basal metazoan phylum, lack true neuronal cell types. As larvae, these animals express photoreactive cryptochromes proposed to mediate phototactic motile behavior and produce post-synaptic machinery (Rivera et al., 2012; Sakarya et al., 2007). Despite their morphological simplicity, the poriferan immune system relies on much of the molecular machinery that is present in the vertebrate innate immunity, including Toll-like receptors (TLRs) and nod-like receptors (NLRs) (Rosenstiel et al., 2009). Notably, a recent transcriptomic study in the Mediterranean sponge (*Aplysina aerophoba*) identified GPCRs among the most differentially regulated transcripts in response to challenge with PAMPs (Pita et al., 2018a) opening up questions as to how neuroimmune responses occur in the absence of bona fide neurons in this taxon.

Placozoans, primitive metazoans that emerged in the Precambrian period, consist of only six somatic cell types and lack organs, neurons, muscle cells, immune cells, or extracellular matrix (Laumer et al., 2018; Smith et al., 2014; Srivastava et al., 2008). Although canonical TLRs and NLRs are absent from placozoan genomes, much of the downstream signaling pathways are present (Kamm et al., 2019). Additionally, the *Trichoplax* sp. genome encodes a diverse array of scavenger receptors (Kamm et al., 2019). Intracellular defenses in this lineage include an expansion in the genes encoding apoptosis inducing factor (Apaf-1), suggesting that apoptotic cell death may be an important component of immune response (Kamm et al., 2019). Despite the absence of neurons, the placozoan genome contains many neuronal genes including SNAP25, SNARE, and 85 GPCRs (Srivastava et al., 2008). Interestingly, GPCRs are evolutionarily ancient, transmembrane proteins that transduce neuropeptide signaling (Jékely, 2013; Schöneberg et al., 2007; Strotmann et al., 2011). Placozoa have been shown to modulate ciliary movement and folding behaviors in response to neuropeptides in their gland cells (Alzugaray et al., 2019; Varoqueaux et al., 2018). Whether placozoans release neuropeptides in response to pathogens is unknown but this kind of experiment may reveal unique neuroimmune pathways and perhaps unveil the most ancient NICUs in the animal kingdom. The diversity of nervous systems in basal metazoans reflects 600 million years of ongoing evolution, and also highlights the evolutionary flexibility of these systems (Yin et al., 2015).

Although cnidarians lack mesoderm, and therefore motile immune cells, searches for innate immune genes reveal that while both most cnidarians have canonical a TLR signaling pathway and complement-effector pathway, these pathways are degenerated in the model cnidarian Hydra (Miller et al., 2007; Brennan et al., 2017). Neuroimmune cooperation has nevertheless mostly been described in Hydra. The Hydra nerve net differentially secretes neuropeptides across the animal’s body that exhibit specific antimicrobial properties to regulate the resident community of symbiotic microbes (Augustin et al., 2017; Klimovich and Bosch, 2018). Using single-cell RNA-Sequencing (sc-RNA-Seq), pacemaker neurons were recently discovered in the head region of the hydra with conserved function and protein machinery to mammalian intestinal cells of Cajal (Klimovich et al., 2020). Interestingly, these neurons express the PRR pathway and many AMPs whose expression, along with pacemaker marker proteins, and contractile function are abrogated when raised under germ-free conditions (Klimovich et al., 2020; Murillo-Rincon et al., 2017). While the precise role of these neuropeptides in regulating the Hydra microbiome and immune system remains unknown, it is clear that, as in mammals, the microbiome is imperative for proper neuronal and immune function in Hydra. Neuroimmune cooperation in corals and anemones, however, remains underexplored.

Taken together, the current body of work highlights our view that understanding neuroimmune communication in basal metazoans is limited to a handful of studies in a small number of taxa and, therefore, gathering new experimental evidence in basal metazoans is key to unveiling new primordial mechanisms by which neurons and immune cells detect and fight danger. Genome editing, single cell, and spatial transcriptomics can now illuminate responses to danger in non-model organisms at unprecedented high-resolution levels. Next, we will take a deep look into the tunicates, a re-emerging group of model organisms, and indicate exciting avenues in NICU research.

## 4.Unique NICUs in tunicates may govern regeneration and colonialism

Tunicates, also known as sea squirts, are benthic marine invertebrates that emerged 550 mya (Lemaire, 2011). Tunicates are the closest relatives to vertebrates (Delsuc et al., 2006). There are approximately 3000 species of tunicates including several model organisms such as *Ciona intestinalis, Halocynthia roretzi*, and *Botryllus schlosseri* (Holland, 2016).

Ascidians, which comprise the largest class of tunicates, have complex developmental life cycles in which embryogenesis results in a chordate tadpole that, after hatching, metamorphoses into a sessile form known as an oozooid that asexually buds to create a colony of adult zooids (Holland, 2016; Kowarsky et al., 2021; Manni et al., 2007). The zooid has a complex body plan with central and peripheral nervous systems, and a complex hematopoietic system with multiple immune cell types (Braun and Stach, 2019; Burighel et al., 2005; Franchi and Ballarin, 2017; Mackie and Burighel, 2005; Rosental et al., 2018). Detailed descriptions of the peripheral nervous system of *Botryllus* reveal that each zooid of the colony possesses an individual nerve plexus that is discontinuous with that of adjacent zooids (Burighel et al., 2005). Furthermore, the dense innervation of the *Botryllus* gut may represent an important site for NICUs; as filter feeders, tunicate guts are regularly exposed to the microbe-rich seawater environment (Utermann et al., 2020). Availability of sterile, or germ-free, tunicate models offers exciting opportunities to interrogate neuroimmune interactions in the context of microbiota recognition and regulation (Leigh et al., 2016).

Colonial tunicates have been long-studied as a model for allorecognition (De Tomaso, 2009; Oka and Watanabe, 1967; Oka and Watanabe, 1960; Oka and Watanabe, 1957; Sabbadin, 1962; Scofield et al., 1982). Colony fusion follows similar principles to vertebrate histocompatibility and only occurs when both colonies share one or more alleles of the polymorphic *Botryllus Histocompatibility Factor* (BHF) gene (De Tomaso, 2009; De Tomaso et al., 2005; Rinkevich et al., 2012; Taketa and De Tomaso, 2015; Voskoboynik et al., 2013). When tunicate colonies with different BHF alleles come into contact, a blood-based inflammatory reaction occurs (Taketa and De Tomaso, 2015). At the point of rejection, cytotoxic blood cells known as morula cells secrete factors that lead to reactive oxidative damage causing cell apoptosis and cytotoxic lesions (Ballarin et al., 2002; Ballarin et al., 1995; Menin et al., 2005; Taketa and De Tomaso, 2015). Cytotoxic natural killer (NK) cells are important innate immune cells involved in allorecognition. A subpopulation of *Botryllus* blood cells express CD94-related transmembrane receptor protein, which is a specific cell surface marker of vertebrate NK cells (Khalturin et al., 2003). Furthermore, cytotoxicity of morula cells is mediated by BHF self-recognition (Rosental et al., 2018). Even after fusion, chimeric colonies can undergo rejection in a process known as allogenic resorption which involves both phagocytic and cytotoxic pathways (Corey et al., 2016). Notably, to facilitate allorecognition reactions, cytotoxic morula cells congregate at the tips of ampullae and exist in the tunic matrix where cilia of primary sensory neurons also project (Mackie and Burighel, 2005; Menin et al., 2005). Although presence of neurons or neuronal derived molecules in the point of allogenic rejection or resorption in tunicates has not been directly observed, it is plausible that they play a role in regulation of cytotoxic events against infection or in homeostasis (Burighel et al., 2001; De Santo and Dudley, 1969; Mackie and Burighel, 2005; Torrence and Cloney, 1981; Zaniolo et al., 2002). Many avenues of study into NICUs in tunicates are open for investigation.

Perhaps the best-characterized tunicate model organism is the solidary ascidian, *Ciona intestinalis*, which is able to regenerate its neural complex (NC) and syphon (Jeffery, 2015; Mingazzini, 1891). Following ablation of the NC, the first step in regeneration is wound healing (Jeffery, 2015). In the course of this process, pluripotent cells that circulate in the vasculature rapidly invade the wound site and, along with other progenitor cells, give rise to the regenerating brain (Bollner, 1989; Bollner et al., 1992; Da Silva et al., 2015; Lender and Bouchard-Madrelle, 1964). Interestingly, in crayfish, adult-born neurons are derived from hemocytes (Benton et al., 2014). Thus, a clear connection exists between immune cells and neurons in the context of invertebrate regeneration that deserves further investigation.

An in-depth, high-resolution developmental and cellular atlas of the immune and nervous system of model tunicates has recently become available (Kowarsky et al., 2021), which should further facilitate investigation of neuroimmune interactions in tunicates. In summary, tunicates harness tremendous biodiversity, unique lifestyles, and regenerative capacities that make them an ideal candidate taxon for the discovery of novel NICUs especially as the closest extant relative to vertebrates with central nervous systems (Holland, 2016).

## 5.Centralization of the nervous system in invertebrates: crosstalk with the immune system

The evolutionary transition from diffuse to centralized nervous systems in Bilateralia is still an open question in neuroscience. The function of the central nervous system (CNS) is to integrate peripheral environmental inputs and orchestrate whole-body downstream behaviors. Although centralization of the nervous system is often considered to be a vertebrate innovation, invertebrates such as echinoderms, arthropods, nematodes, molluscs, and annelids have a CNS with various shapes and degrees of complexity in different animal phyla (Arendt et al., 2008; Figure 2). Whether the vertebrate and invertebrate CNS trace back to a common ancestor or evolved independently is still unknown. Conservation of brain patterning mechanisms suggests the mechanisms for centralization of nervous systems may be as ancient as the last common bilaterian ancestor (Denes et al., 2007; Hartenstein and Stollewerk, 2015; Telford, 2007). Interestingly, current evidence suggests that glial cells, also present in invertebrates, have arisen multiple times independently (Hartenstein and Giangrande, 2018). Despite the morphological disparities between the cnidarian nerve nets, the ventral central ganglia in arthropods and the vertebrate brain, the underlying function of nervous systems mirrors that of immune systems: sensing the environment and responding appropriately to maximize survival.

Findings from protostomes suggest that connections between CNS and immune functions are widespread among Metazoa. *Drosophila* neurogenesis has been studied in great detail and exhibits many similarities to mammalian models (Buescher et al., 2002; Grens et al., 1995; Hartenstein and Stollewerk, 2015; Heitzler et al., 1996). For example, the cell adhesion molecule *dscam* is involved in axonal segregation in both *Drosophila* and mammals. However, this molecule has been co-opted into a unique immune function in insect hemocytes (see Table 1 and section 8.1) (Goyal et al., 2019; Hattori et al., 2009; Ng and Kurtz, 2020). Additionally, a *Drosophila* cytokine and its receptor signaling through the JAK/STAT pathway are necessary for long-term odor memory (Copf et al., 2011). To date, few studies have investigated changes in *Drosophila* CNS gene expression after exposure to pathogens or PAMPs, however, the identity and homeostatic function of glial cells are becoming elucidated (Yildirim et al., 2019). Non-model insect species have also revealed interesting neuroimmune connections. For instance, the kissing bug produces a suite of CNS neuropeptides that, in addition to their roles in brain development and function, may have antimicrobial activity (Ons et al., 2009).

Annelids also have a CNS in the form of a ventral nerve cord and a prostomial brain. Evolutionary conserved mechanisms of neuroimmune communication have been unraveled in earthworms (Oligochaeta). For instance, the coelomic cytolytic factor, a PRR in annelids which is similar to mammalian TNF, appears to interact with ion channels present on the macrophage cellular membrane, depolarizing the cell and triggering release of pro-inflammatory cytokines (Bilej et al., 2006). Whether this PRR is expressed on annelid neurons in the CNS regulating immunity remains to be elucidated. The annelid *Hirudo verbena* (Hirudea), or medicinal leech, has also emerged as a model for neuroimmune studies in invertebrates (Tasiemski and Salzet, 2010). *H. verbena* has a segmented, ventral CNS that regenerates upon injury. Microbial exposure increases AMP synthesis by neurons and microglia and results in accelerated repair of the leech CNS upon injury (Schikorski et al., 2008). Neuronal expression of PRRs and AMPs after exposure to bacteria and the viral mimic poly(I:C) was also shown in the leech CNS (Tasiemski and Salzet, 2017). Combined, these works underscore the need for effective innate immune response to ensure CNS function in health and disease in invertebrates. Finally, neurobiology studies on the marine annelid *Platynereis dumerelii* (Polychaeta) have revealed fascinating aspects of CNS evolution and neuropeptide function. For instance, the myoinhibitory neuropeptide (MIP) produced by neurosecretory cells in the animal’s brain controls larval settlement (Conzelmann et al., 2013). Unfortunately, the immune system of *P. dumerelii* has not been studied in detail and therefore CNS immune responses remain essentially unknown. Collectively, the current body of work offers speckled, intriguing insights as to how invertebrate CNS structures and hemocytes cooperate to optimize danger detection and behavioral avoidance to pathogens; the breadth of invertebrate species leaves much to be explored in the neuroimmunology field. Mapping where NICUs are located in these taxa should reveal evolutionary conserved connections between the CNS and the innate immune system.

Cephalochordates, commonly known as amphioxus, are basal chordates that possess a primitive brain that is homologous to the vertebrate hindbrain but lack a telencephalon (Feinberg and Mallatt, 2013). Amphioxus lacks an adaptive immune system but does possess genes that support the adaptive immune system (Feinberg and Mallatt, 2013; Holland, 2009; Sugahara et al., 2017; Yu et al., 2005). Like invertebrates, amphioxus PRRs are expanded (Huang et al., 2008). Bulk RNA-seq analysis of amphioxus after intraperitoneal injection with lipopolysaccharide (LPS) identified several neuronal related genes. For instance, spondin, a gene involved in axonal guidance, was one of the most down regulated genes, whereas nerve growth factor receptor was one of the most upregulated genes 24 hr after LPS injection (Zhang et al., 2017). Furthermore, the neurogenesis-related notch KEGG pathway was enriched 6 hr after injection and the olfactory transduction KEGG pathway was enriched 48 hr after injection (Zhang et al., 2017). Combined, this study suggests neuronal participation in the amphioxus innate immune response and motivates the search for NICUs in cephalochordates. Such studies could elucidate unique NICUs that predate the emergence of the adaptive immune system and vertebrate CNS structures such as the telencephalon.

## 6.Centralization of the nervous system in vertebrates and the immunological big bang: a case for investigating agnathan neuroimmune interactions

Although centralization of the nervous system is found in protochordates, two major evolutionary innovations occurred in the nervous and immune systems coincident with the emergence of agnathan vertebrates (~500 mya): the emergence of a telencephalon as part of the CNS and the appearance of an adaptive immune system (Guérin et al., 2009; Holland, 2015; Figure 2). While agnathans possess a nervous system analogous to jawed vertebrates, this lineage evolved an adaptive immune system based on variable lymphocyte receptors (VLRs) that is functionally analogous to the Rag1/2-mediated, immunoglobulin-based adaptive immune system (Flajnik, 2014; Sugahara et al., 2016).

Within both vertebrate lineages, adaptive immune systems are based on lymphocyte receptors that somatically recombine to generate nearly infinite epitope recognition. Prior to the divergence of echinoderms and chordates, a transib-like transposon inserted into the genome of a common ancestor to form the Rag1/2 complex (Fugmann et al., 2006; Huang et al., 2016; Zhang et al., 2019b). Within jawed vertebrates, two lineages of mesodermally derived lymphocytes (B and T cells) co-opted the RAG1/2 transposases to rearrange V(D)J gene segments and create complementarity determining regions (CDRs) with clonally unique epitope specificity (Bassing et al., 2002). Notably, one of the fundamental tenets of adaptive immune systems is self-tolerance: somatically recombined receptors that bind host proteins are deleted during lymphocyte development. This process is mediated by proteins within the MHC and has been proposed to have been co-opted from an ancient olfactory system still used in mate choice based on peptide carrier protein phenotype (Andreou et al., 2017; Boehm, 2006; Penn and Potts, 1998). Even after negative selection to eliminate receptors that bind self-antigens, the adaptive immune system is a powerful, lethal tool in vertebrates that requires tight regulation. This regulation, as described in section 9.3, is partly mediated by the nervous system.

In parallel with the Rag1/2-based adaptive immune system of jawed vertebrates, jawless vertebrates convergently evolved a distinct type of adaptive immunity (Flajnik, 2014). Here, VLRs exist as gene segment clusters that are re-arranged in lymphocyte-like cells by orthologs of activation induced cytidine deaminase (AID), known as CDA1/CDA2 (Rogozin et al., 2007). Transcriptional profiles suggest that the two independent mechanisms of lymphocyte receptor generation arose after the origin of three lymphocyte populations: a B cell-like progenitor, and two subsets of T-cell progenitors. BCRs and VLRB proteins are expressed as membrane-bound receptors that are secreted after activation. The membrane-bound VLRC and γδ TCR are expressed on cells that localize in skin (Flajnik, 2014; Hirano et al., 2013). Finally, T cells (both VLRA^+^ and VLRC^+^) develop in the FoxN1^+^ agnathan thymoid, suggesting that VLR cells follow similar regulation mechanisms of gnathostome adaptive immunity (Bajoghli et al., 2011). Lamprey CNS patterning during development mirrors that of gnathostomes, but whether the agnathan CNS and meninges mount immune responses against local or distant danger is essentially unknown (Sugahara et al., 2016). Furthermore, cross-regulation between the agnathan nervous and immune systems remains unexplored. Recently however, CRISPR-based genome editing was used successfully in lampreys to eliminate CDA2, the enzyme that mediates VLRB rearrangements (Morimoto et al., 2020). Consequently, this model system is now ripe for the investigation of changes in the nervous system in the absence of one arm of the immune system. Thus, investigation of neuroimmune interactions in the agnathan CNS with a convergent, yet unique, adaptive immune system is an exciting avenue for this field.

## 7.Draining the brain: evolution of brain lymphatics

As the vertebrate CNS became larger and more compartmentalized, increased metabolic waste and immunological demands necessitated a waste removal system to avoid deleterious effects on neuronal function (Kanamori and Kipnis, 2020; Louveau et al., 2018). Lymphatic vessels present in the meninges of the CNS of mammals drain molecules and immune cells from the subarachnoid space into the cervical lymph nodes (Aspelund et al., 2015; Da Mesquita et al., 2018; Louveau et al., 2015). Beyond creating an avenue for immune surveillance of antigens in the brain, meningeal lymphatics have been implicated in neuroinflammatory disease (Da Mesquita et al., 2018; Louveau et al., 2015).

Although first discovered in mice, recent studies have revealed the presence of brain lymphatic vessels in zebrafish, a cold-blooded vertebrate (Bower et al., 2017; Castranova et al., 2021; Chen et al., 2019b; van Lessen et al., 2017). Bony fish have a similar innate and adaptive immune system to mammals, yet a smaller CNS and lower mass-specific metabolic rates compared to those of mammals. The presence of a meningeal lymphatic system clearing macromolecules seems to be required in both fish and mammals for adequate function of the CNS although whether it was a requirement for existence of a complex CNS is unclear (Louveau et al., 2015; van Lessen et al., 2017). Agnathan vertebrates have a CNS and meninges, but the existence of lymphatics, and therefore a lymphatic system, associated with the CNS is unknown. Furthermore, based on current knowledge, the emergence of brain lymphatics coincides with the emergence of B- and T-cell immunity in jawed vertebrates, though whether the existence of dangerous lymphocytes obliges lymphatic evolution to protect the vulnerable CNS is unknown (Figure 2). Future studies in agnathans and invertebrates with centralized nervous systems, such as *Drosophila*, may reveal unique or conserved systems to drain the CNS in organisms where B and T cells are not present.

## 8.Genes and gene families that mediate both neuronal and immune functions

Given their similar functions, it is not surprising that the immune and nervous systems rely on similar proteins and protein domains (Table 1). Leucine-rich repeat (LRR) domains detect pathogens as part of PRRs and VLRs and guide neuronal development (Slit proteins and LRRK). Similarly, the immunoglobulin (Ig) domains that form the basis of jawed vertebrate adaptive immunity (i.e., BCR, TCR, MHC) also regulate neuronal development and synapse formation (Sanes and Zipursky, 2020). Here, we explore three examples of gene families employed by both the nervous and immune systems but many more exist, including cytokines, neurotrophins, perforins, and complement (Table 1; Kolosowska et al., 2019; Minnone et al., 2017; Ni and Gilbert, 2017; Stevens et al., 2007; Yang et al., 2016). Notably, many of these play crucial roles in the development of both the nervous and immune systems.

### 8.1 DSCAM

The Down syndrome cell adhesion molecule (DSCAM) is a member of the Ig superfamily expressed on the cell surface. In both mammals and arthropods, DSCAM regulates self-avoidance during neural development (Garrett et al., 2018; Hattori et al., 2009). That is, if two dendrites from a single neuron encounter one another during morphogenesis, they repel each other, preventing inappropriate synapse formation and leading to even spatial distribution. In this context, DSCAM appears to operate in concert with two cell adhesion molecules: cadherins and protocadherins (Garrett et al., 2018). Evolutionary analysis suggests that although the *dscam* gene is present throughout Bilateria, Pancrustacea (insects and crustaceans) exhibit a distinct form of isoform diversification (Armitage et al., 2012). While mammalian neurons only express a single *dscam1* transcript, arthropods have evolved a unique evolutionary strategy in which a single *dscam* gene is capable of producing over 38,000 isoforms due to mutually exclusive alternative splicing (Brites and Du Pasquier, 2015). In *Drosophila*, the *dscam* gene consists of 22 exons, of which four are clusters of tandemly arrayed sequences. All but one of these arrays are spliced out during transcript processing. Stochastic expression of these hypervariable isoforms within individual neurons drives the self-avoidance behavior.

Although mammalian *dscam1* expression is restricted to neurons, *dscam* homologs play a unique role in the crustacean immune system (Ng and Kurtz, 2020; Table 1). Isoform variability was originally described in transcripts isolated from *Drosophila* hemocytes (Watson et al., 2005). Numerous reports have investigated transcriptional changes in the *dscam* isoform repertoire after pathogen challenge in a variety of insect and crustacean species (Ng and Kurtz, 2020). Furthermore, in crabs DSCAM acts as an opsonin; specific isoforms were shown to bind bacteria and promote phagocytosis (Li et al., 2018). In contrast to the neural expression, where DSCAM hypervariability serves to avoid self, within hemocytes, diverse DSCAM proteins function to bind to a wide array of pathogens. Interestingly, the expression of pathogen-specific isoforms can be upregulated in response to immune challenge (Li et al., 2018). The hypervariable family of *dscam* transcripts thus highlights how the immune and nervous systems in Pancrustacea deploy a single mechanism for generating diversity with distinct end goals.

### 8.2 Toll-like receptors

TLRs are prototypic PRRs with fundamental roles in animal innate immune responses. The protein Toll was originally described as a regulator of dorsoventral patterning in early *Drosophila* embryogenesis. However, it was observed that flies with loss-of-function mutations in the Toll signaling pathway exhibited impaired immune responses (Lemaitre et al., 1996). Subsequently, genetic and functional assays implicated a mammalian homolog of Toll (Toll-like receptor 4; TLR4) as a receptor for LPS (Poltorak et al., 1998). Most vertebrate genomes contain 10–20 TLR paralogs that recognize PAMPs, and independent expansions of TLRs occurred several times in invertebrate lineages (Buckley and Rast, 2015). TLRs are central to innate immunity and activate adaptive responses although they are also expressed by many cell types outside the immune system including epithelial cells, endothelial cells, and neurons. As transmembrane receptors, TLRs localize to either the cell surface, where they recognize PAMPs (e.g., LPS, flagellin) or endosomes where they largely detect nucleic acids (Kawai and Akira, 2010). Ligand binding to the TLR ectodomain, composed of LRRs, initiates a signaling pathway that culminates in NF-kB activation. This highly conserved pathway is present throughout Metazoa, including poriferans (Figure 2).

Surprisingly, TLR knock-out studies in mice revealed not only impaired immune responses, but also neural phenotypes. Of the 13 TLRs in mice, five are expressed within the CNS, where they have roles in both neuronal development as well as neural plasticity, cognition, and behavior (Morimoto and Nakajima, 2019). TLR2, TLR3, and TLR4 have been shown to regulate proliferation in neural progenitor cells (NPCs) (Chen et al., 2019a; Lathia et al., 2008). TLRs 3, 7, and 8 also regulate synapse formation (Hung et al., 2018). During brain development, synapse formation is activity dependent, leading to death of inactive axons, which creates apoptotic bodies and releases nucleic acids that activate TLR responses (Ma et al., 2006; Zhang and Poo, 2001). It has been suggested that these responses induce cytokine expression, which subsequently attracts phagocytic microglia (Chen et al., 2019a; Ma et al., 2006). While nucleic acid sensing TLRs are typically restricted to endosomes in the nervous system, these TLRs are expressed on cell surfaces, where they detect self-antigens. This conserved detection and signaling pathway thus represents a fundamental aspect of neuroimmune communication.

### 8.3 Olfactory receptor superfamily

The olfactory receptor (OR) superfamily is a diverse group of rhodopsin-type GPCRs that detect chemosensory cues in the environment. Mammalian genomes typically contain very large (~1,000) numbers of OR genes (Niimura, 2012). Rhodopsin-type GPCRs emerged 580–700 mya and are present in Porifera and Placozoa (Churcher and Taylor, 2011). For several OR groups, orthologs can be clearly identified in both chordates and cnidarians (Churcher and Taylor, 2011). Frequent duplication and divergence within the OR gene family early in metazoans as well as the role of rhodopsin-type GPCRs in brain development and synapse formation suggest that ORs were essential in the emergence of the CNS (Churcher and Taylor, 2011). Similarly, in mammals, OR expression in olfactory sensory neural axons is necessary for axonal outgrowth to discrete glomeruli in the olfactory bulb (Feinstein et al., 2004).

Within immune cells, ORs detect pathogens and pathogenic products (Li et al., 2013). Moreover, OR expression in mucosal lymphoid tissues may contribute to tissue organization; recent transcriptomic analyses reveal the enrichment of genes within the olfactory pathway in lymph nodes and Peyer’s patches of mice suggesting co-option of ORs in mammalian lymphoid structures (Heimroth et al., 2020; Table 1). Mammalian lymph nodes are innervated by both sympathetic and sensory neurons, including peptidergic neurons capable of regulating gene expression in endothelial, stromal, and innate immune cells (Huang et al., 2021). Given these dual roles in the nervous and immune systems, the original OR function remains unclear. It is clear, however, that the bidirectional neuroimmune communication in lymphatic tissues mediated by ORs is highly sophisticated and occurs both during development and under steady-state conditions (Huang et al., 2021).

## 9.Bringing neurons into the defense game

**Figure 3.**
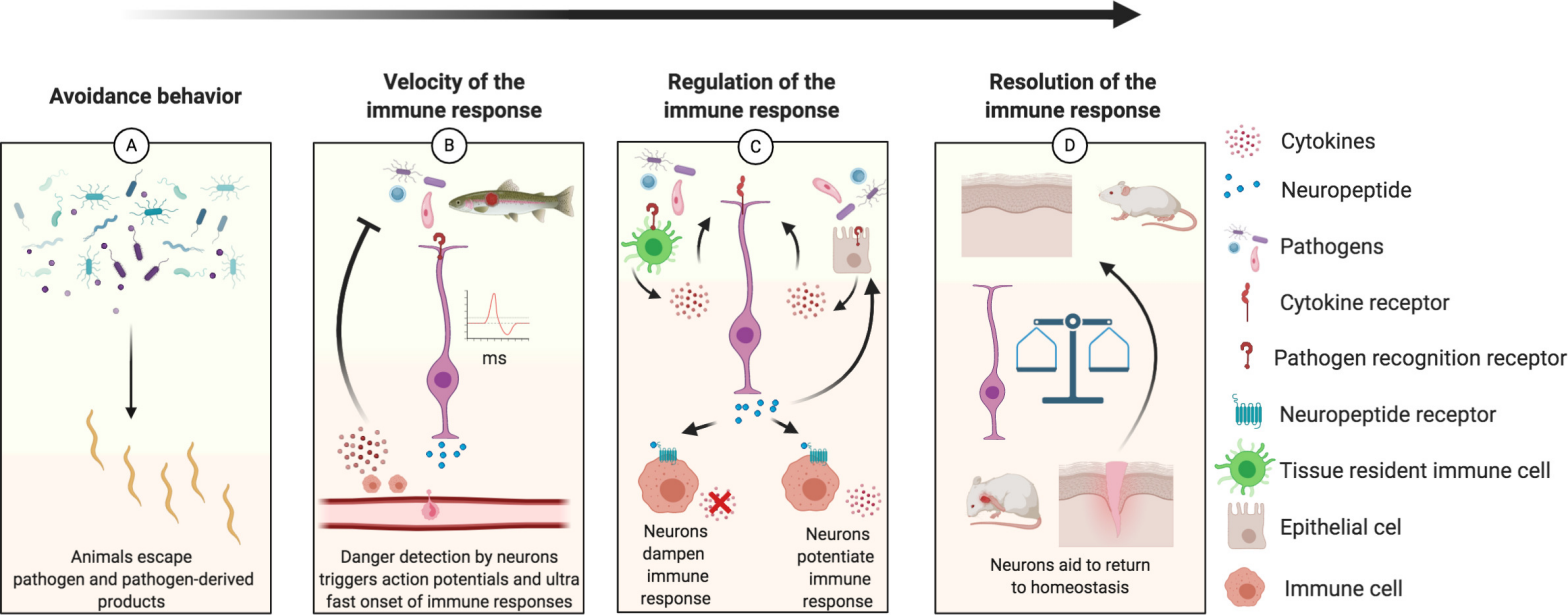
Schematic diagram illustrating how neurons play a role in the regulation of each step of the immune response, from inception to resolution. (**A**) Sensory neurons detect danger cues that are then transduced to move the animals away from potential deleterious infection. For example, in *C elegans npr-1* expressing neurons detect oxygen levels to avoid harmful pathogens (Hoffman and Aballay, 2019; Singh and Aballay, 2019; Styer et al., 2008). Furthermore, bacterially infected female *Drosophila* will lay fewer eggs (Kurz et al., 2017; Masuzzo et al., 2019). (**B**) Sensory neurons directly detect pathogen presence resulting in rapid action potentials and release of neurotransmitters that increase the velocity of an immune response. Neurons ultra-rapid communication to immune cell reservoirs deploy immune cells quicker than chemokine signals; such as recruiting CD8^+^ cells to the olfactory organ after detection of neurotrophic virus by sensory neurons (Chavan and Tracey, 2017; Sepahi et al., 2019). The immune responses triggered by neurons can be biased to be neuroprotective while dealing with infection such as in the myenteric plexus of the gut where ﻿b2 adrenergic receptor signaling polarizes muscularis macrophages to express *Arg1,* associated with an M2 phenotype (Gabanyi et al., 2016; Lai et al., 2020). (**C**) Pathogens detected by immune and epithelial cells provoke release of cytokines and other immune factors that are pro-inflammatory and potentially deleterious to the tissue if allowed to propagate inflammation without regulation. Local neurons are rapidly activated by the inflammatory environment and PAMPs, releasing neurotransmitters that tune the immune response (immune cells and epithelial cells) toward a less pro-inflammatory phenotype (Baral et al., 2018; Basbaum et al., 2009; Cardoso et al., 2017; Chu et al., 2020; Labed et al., 2018; Ramirez et al., 2020). A specific example is neuromedin U that binds to its cognate receptor on ILC2s enhancing their type 2/repair phenotype mediating protection against worm infections (Cardoso et al., 2017). Another example includes the reduction of systemic inflammation when the vagus nerve is stimulated (Borovikova et al., 2000). However, suppressive neuronal responses can be detrimental and favor infection as when TRPV1 neurons release CGRP and suppress neutrophil recruitment, leading to *S. pyogenes* lesion progression compared to animals lacking CGRP signaling (Pinho-Ribeiro et al., 2017; Chu et al., 2020). (**D**) The immune system and nervous system must balance their responses over time to return to homeostasis (Aurora and Olson, 2014; Veiga-Fernandes and Artis, 2018). An initial immune response is necessary for clearance of pathogen and debris and to initiate growth factor responses in neurons, but prolonged inflammation can be detrimental to neuronal regeneration as seen in the olfactory system and spinal cords with NF-κb/chemokine and TNFα signaling, respectively, leading to loss of regenerative abilities (Chen et al., 2019a; Godwin et al., 2013; Tsai et al., 2019; Tsarouchas et al., 2018). Another example includes the resolution of inflammation controlled by the sympathetic nervous system in mice (Körner et al., 2019).

Sensory neurons detect environmental conditions and transmit afferent information to an integration hub (the CNS in vertebrates), which subsequently sends efferent signals to extremities to coordinate a response. When a pathogen is detected by a sensory neuron, the efferent signals elicit both overt avoidance behaviors and prime immune responses to protect the organism from infection or deleterious inflammation. Here we propose several ways by which the immune response benefits from neuronal activity (Figure 3). Whereas some may be restricted to specific metazoan lineages, many of these beneficial interactions are likely widely conserved.

### 9.1 Pathogen avoidance

The ultimate protection mechanism is to avoid pathogens altogether. Although the neuroethology of how animals integrate sensory cues from pathogens to modulate behavior remains largely unknown, every animal interrogated thus far, particularly highly social animals, displays some form of avoidance behavior (Curtis, 2014; Figure 2). Generally, animals avoid individuals of their own species that are sick. In rodents, avoidance behaviors are mediated by the vomeronasal system and specific synchronization between the ventral hippocampus and the medial prefrontal cortex; mice with ablated vomeronasal organs no longer avoid odors from parasitized mice (Boillat et al., 2015; Padilla-Coreano et al., 2019; Padilla-Coreano et al., 2016).

Avoidance behaviors have also been observed in other jawed vertebrates (e.g., bony fishes), agnathans, and invertebrates (Behringer et al., 2018). Interestingly, germ-free zebrafish larvae have been found to be hypermobile and exhibit increased anxiety-like behaviors compared to conventionalized larvae, demonstrating that behavioral circuits require the presence of a microbiome to develop properly (Davis et al., 2016). The agnathan sea lampreys display avoidance behaviors to environmental chemosensory cues such as alarm odors secreted from heterospecifics; however, avoidance behavior to pathogens or infected lampreys has not been investigated to date (Hume and Wagner, 2018).

While such studies clearly connect behavior to nervous system function, understanding how afferent sensory cues are integrated in response to danger is far more complicated. However, smaller, less-complex model organisms may offer some answers. In *Drosophila*, during bacterial infection, peptidoglycan-NF-κb signaling specifically activates a subset of octopamine neurons and changes egg-laying physiology of females (Kurz et al., 2017; Masuzzo et al., 2019). Additionally, both *Drosophila* gustatory and olfactory neurons have been found necessary and sufficient to avoid LPS and geosmin, respectively (Soldano et al., 2016; Stensmyr et al., 2012). Furthermore, *C. elegans* sensory neurons mediate avoidance behaviors in response to pathogen-contaminated food, thereby lowering pathogen burden (Anderson and McMullan, 2018; Styer et al., 2008). However, *C. elegans* also display avoidance behavior to ﻿*Bacillus anthracis* despite the fact that this bacterium is not harmful (Turner et al., 2020). Individual worms learn to avoid pathogenic *Pseudomonas aeruginosa* after luminal bloating alters the TGF-β/DAF-7 pathway and activates the GPCR NPR-1, causing aerotaxis away from an oxygen-deficient bacterial lawn (Singh and Aballay, 2019). Interestingly, *C. elegans* pathogen-specific avoidance behaviors can be transgenerational, a phenomenon mediated by two immunological pathways: TGF-β signaling in sensory neurons and the Piwi/Argonaute small RNA pathway (Moore et al., 2019). Behaviors that avoid wounding and infection from pathogens are key to survival because once barriers are breached, immune responses are energetically costly and may leave long-lasting damage in the host.

### 9.2 Velocity of the immune response

When behaviors fall short, barrier tissues provide the next line of defense against pathogens. When pathogens breach barrier tissues, innate immune cells are recruited by chemokines secreted by epithelial cells who sense PAMPs and damage-associated molecular patterns (DAMPs). Innervating neurons sense microorganisms, and their products, directly and influence the direction of immune responses (pro- or anti-inflammatory) several orders of magnitude faster than those induced in immune cells (Chavan and Tracey, 2017; Chiu et al., 2013; Foster et al., 2017; Pinho-Ribeiro et al., 2017). Pathogen detection by neurons results in rapid electrical activation (within milliseconds) and release of chemical mediators that either enhance or suppress the local and distant immune response (Chu et al., 2020) (Figure 3). Rapid neuronal responses offer clear advantages for the host, thereby offering positive pressures for the immune system to gain expression of receptors for neuronal messengers as an additional, accelerated mechanism of pathogen surveillance.

Recent studies highlight anticipatory immunity by mammalian nociceptive TRPV1^+^ neurons. Activated neurons mediate a type-17 immune response in contiguous areas resulting in protection of regional areas of the skin from subsequent fungal and bacterial infection (Cohen et al., 2019). On an organismal level, electrical activation of dopaminergic and cholinergic neurons in the vagus nerve is sufficient to regulate hematopoietic tissues such as the spleen and can inhibit pro-inflammatory cytokine secretion (Chavan and Tracey, 2017; Kressel et al., 2020). In the case of the olfactory-CNS axis, pathogen activation of olfactory sensory neurons (OSNs) quickly elicits neuroimmune responses in the CNS. This was recently demonstrated in a bony fish model, in which electro-olfactograms show that trout OSNs detect rhabdovirus and become electrically activated (Sepahi et al., 2019). Concomitantly, within minutes of nasal delivery of rhabdovirus, antiviral immune responses occur in the olfactory epithelium as well as the CNS (Sepahi et al., 2019). This response, however, requires viral sensing by the OSNs, highlighting that ultra-rapid coordination of immune responses by neurons is critical in chemosensory organs.

Although the concept of neuronal speed in the context of the immune response has not been widely discussed in the mammalian literature, we propose that neuronal innervation of lymphoid tissues as well as the wide presence of NICUs at mammalian tissue barriers may have been evolutionary driven to optimize the speed of the immune response at the organismal level. Furthermore, increased immune speed can be beneficial to the neurons themselves. In the gut, enteric bacteria activate sympathetic and nociceptive neurons that, in turn, polarize muscularis macrophages towards a tissue protection profile that protects neurons from dying 7 days after infection (Gabanyi et al., 2016; Lai et al., 2020; Matheis et al., 2020). Neurons directly sense danger, and their action potentials release immunogenic neurotransmitters that affect immune system function that can be protective of the organism and neurons themselves. However, as infection progresses, regulation of the immune response by neurons must be balanced for pathogen clearance and optimal host survival.

### 9.3 Adding layers of regulation to the immune response

Upon binding PAMPs and/or DAMPs, PRRs initiate cytokine expression. In turn, the cytokine milieu promotes optimal immune responses while dampening others (e.g., humoral vs cellular immunity). Classically, cortisol and the vagus nerve are known to maintain homeostasis and decrease inflammatory pathways to prevent damage (Borovikova et al., 2000). From these studies, it was suggested that the nervous system acts as a final layer of regulation over the mammalian immune system by coordinating large scale-physiological responses (Eberl and Pradeu, 2018). However, viewing neuronal control of inflammation as culmination of regulation neglects experimental evidence demonstrating that instantaneous neuronal control of local immune responses provides the first layer of immune regulation. We propose that neurons impart checkpoints at each step of the defense response, from its inception to resolution and healing (Figure 3).

Neurons are powerful immune suppressors. For example, in a murine model of *Staphylococcus aureus* pneumonia, ablation of TRPV1^+^ nociceptive neurons increases neutrophil surveillance and γδ T-cell numbers in the lung, leading to increased survival and bacterial clearance (Baral et al., 2018). A *﻿Citrobacter rodentium* infection model found increased colonic bacterial burdens and T-cell numbers in TRPV1^+^ nociceptor ablated animals compared to controls (Ramirez et al., 2020). Furthermore, infection burden with ﻿*N. brasiliensis* can be experimentally decreased in the gut in the presence of exogenous neuromedin U (Cardoso et al., 2017). This molecule is normally secreted by enteric neurons and enhances immune responses by binding to innate lymphoid cells, (Cardoso et al., 2017). Finally, brainless Xenopus larvae exhibit increased susceptibility to bacterial infection with *E. coli* and an overall increase in myeloid lineage cells and neurons (Herrera-Rincon et al., 2020). In these studies, brains were removed prior to the developmental maturation of the adaptive immune system, so phenotypic differences resulted from neuronal effects on innate immunity (Herrera-Rincon et al., 2020). Therefore, the inhibitory signals from peripheral neurons may prevent deleterious off-target host damage due to the immune response but also affect infection outcomes depending on the pathogen.

Neuronal suppression of the immune system is deeply rooted in animal evolution. In addition to the vertebrate models described above, studies in protostome lineages also highlight the crosstalk between the neural and immune systems. Upon stimulation, *Drosophila* sensory neurons deploy TGF-β to stimulate hemocyte proliferation and adhesion (Makhijani et al., 2017; Table 1). In *C. elegans*, npr-1^+^ neurons suppress the unfolded protein response (UPR), an important innate immune mechanism, thereby increasing pathogen susceptibility (Hoffman and Aballay, 2019; Sun et al., 2011). Additionally, neurons in *C. elegans* release neurotransmitters (e.g., insulin, neuropeptides, octopamine, dopamine, acetylcholine) to modulate pathways involved in host defense in the gut endothelium (e.g., p38 MAPK, UPR, and WNT signaling) (Wani et al., 2020). For instance, *C. elegans* neurons release acetylcholine in response to infection, and acetylcholine then activates the canonical Wnt pathway in intestinal epithelial cells resulting in innate immune responses (Labed et al., 2018). The broad phylogenetic distribution of these findings suggests that neuronal regulation is a fundamental mechanism to prevent collateral damage from the immune response. When unavoidable, the immune and nervous systems mount a coordinated response to repair damage.

### 9.4 Cooperation of neurons and immune cells in animal regeneration

In extreme cases of disease or injury, animals undergo whole-body or limb regeneration. Regenerative capacity varies considerably among animal phyla and depends on interactions between the immune and nervous systems (Bloom, 2014; Cary et al., 2019; Lange and Brand, 2020). In the well-characterized newt regeneration model, phagocytic macrophages are recruited within one day post-amputation; macrophage depletion results in decreased expression of regenerative genes and slows regrowth (Godwin et al., 2013). sc-RNA-Seq analyses confirm this important role for macrophages immediately following amputation and reveal the presence of T cells in the blastema, although the function of these adaptive immune cells is unknown (Leigh et al., 2018). Fluorescent activated cell sorting of early progenitors and RNA-Seq reveal that early dividing cells at the amputation plate recruit myeloid cells through IL-8 signaling, without which the blastema forms but does not regenerate the limb (Tsai et al., 2019). In addition to immune cells, nerves provide neurotrophic factors that are necessary for limb reformation (Wang et al., 2000). Notably, nerve growth factor pathways are suppressed in early blastema progenitors just 4–5 days post-amputation, suggesting that, prior to regeneration, wound healing and developmental patterning must occur (Tsai et al., 2019). The cooperation between nerves and immune cells precisely coordinates the timing and activation of newt limb regeneration, although it has been proposed that that salamanders preserve these regenerative abilities because they have a weaker, more simple immune system (Aurora and Olson, 2014).

Most mammalian spinal cords and CNS heal poorly after injury with the exception of the neonatal opossum and fetal rat (Wheaton et al., 2020). However, more ancient vertebrates such as lampreys maintain regenerative capacity (Bloom, 2014). Excessive influx and pro-inflammatory signatures of oligodendrocytes, macrophages, and microglia (gliosis) may limit regeneration in mammalian spinal cord injuries (Vitalo et al., 2016). A teleost model of spinal cord injury could not recapitulate the gliosis seen in mammals, although glial cells did participate in regeneration (Vitalo et al., 2016). Zebrafish mutants that lack macrophages are unable to heal spinal cord injuries and exhibit prolonged pro-inflammatory states (Tsarouchas et al., 2018). In mice, neuron-derived CCL2 acts as a macrophage chemoattractant; overexpression increases the M2/regenerative macrophage phenotype, leading to increased neurite outgrowth and neuronal regeneration (Kwon et al., 2015).

In mammals, the greatest capacity for neuronal regeneration lies in the olfactory system (Durante et al., 2020). At the basement of the olfactory epithelium, olfactory progenitor cells, known as horizontal basal cells, regenerate olfactory sensory neurons to maintain homeostasis after damage via acute inflammation induction of NF-κB (Chen et al., 2017; Peterson et al., 2019). However, chronic inflammation with prolonged NF-κB activation and CCL19/CCL20 secretion causes increased inflammatory phenotypes of resident immune cells and diminishes the regenerative capacity of the olfactory epithelium (Chen et al., 2019a). Understanding the regulation of inflammation in this naturally regenerating neuroepithelium may help uncover therapeutics; however, more work is needed to understand neuroimmune communication in the olfactory system.

## 10.Conclusions

The nervous system is an invaluable ally for the immune system to fight infection from the early detection of danger to the shutdown of the immune response. Current knowledge on neuroimmune communication is mostly limited to mammalian studies; however, the most fundamental elements that enable this interaction are found deep in the metazoan tree of life. We argue that interrogating the basal branches of the metazoan tree in the context of danger detection using new approaches such as single-cell transcriptomics and CRISPR can illuminate the origins and fundamental principles that govern NICU development and function across taxa.
